# The Party Should Not Last That Long

**DOI:** 10.19102/icrm.2023.14013

**Published:** 2023-01-15

**Authors:** James A. Reiffel

**Affiliations:** ^1^Department of Medicine, Division of Cardiology, Columbia University, New York, NY, USA

**Keywords:** Atrial fibrillation, lone AF, rhythm control, therapy

## Abstract

Too much food, too much wine, and too many friends. You’ll pay the price tomorrow; you shouldn’t have let the party last so long. This analogy seems apt with respect to our new understanding of atrial fibrillation (AF) and approaches to AF. The keys to understanding recent advances in the management of AF and improving outcomes on therapies are an appreciation that: (1) AF is often a progressive disorder; (2) its progression is related to the degree of atrial myopathy that is present; (3) atrial myopathy is a consequence of the effects of underlying comorbidities as well as the effect of AF itself (tachycardic effects on the atria); (4) adverse outcomes can be a consequence of AF, the underlying atrial myopathy, as well as direct consequences of any comorbidities present; (5) rhythm control of AF early in its course as well as early and optimal treatment of underlying comorbidities have been associated with improved outcomes (eg, lower mortality, lesser thromboembolism, lesser heart failure, fewer hospitalizations) in recent trials; (6) therapies not available 2 decades ago during the rate- versus rhythm-control trials have played a role in the new treatment approaches and make the old idea that rate control is as good as rhythm control somewhat obsolescent; and (7) these now indicate that optimal and early rhythm control and comorbidity treatment provide the best results for AF patients.

Too much food, too much wine, and too many friends. You’ll pay the price tomorrow; you shouldn’t have let the party last so long. This analogy seems apt with respect to our new understanding of atrial fibrillation (AF) and approaches to AF. Moreover, with the advent of modern monitoring devices such as smartphones, interrogatable pacemakers/defibrillators, and insertable cardiac monitors able to reveal subclinical AF, many patients with AF do not even know when the party began.

AF is one of the scourges of modern mankind—or so it seems. Patients with AF are increasing in number significantly (due to older populations, greater survival with cardiovascular disorders, and enhanced diagnostic modalities) and can experience annoying symptoms, such as palpitations, dizziness, or discomfort, that reduce the daily quality of life. However, its major associated adverse outcomes, including stroke, heart failure, and death, are what we fear the most. Significantly, and often under-considered, these are largely dependent on the company AF keeps rather than just on AF alone and the synergistic effect between AF and these other conditions **(Figure 1)**.

In the absence of advanced age or clinically evident comorbidities (eg, hypertension, heart failure, vascular disease, diabetes, obesity), AF has been termed “lone AF.” “Lone” AF carries a significantly lower risk for mortality, heart failure, thromboembolism, or hospitalization compared to AF with associated comorbidities.^1^ Confoundingly, the definition of “lone” AF is uncertain, as, when the term was introduced almost 70 years ago, precipitants now recognized as important in some patients, such as genetics, alcohol, excessive vagotonia, obesity, sleep apnea, “recreational drugs,” and more, were not appreciated and/or not sought in patients with seemingly “lone” AF.^2–5^ Importantly, these factors vary among “lone” AF patients and can affect their clinical course, thus making the actual prognosis of “lone” AF uncertain. Nonetheless, it is clear that being diagnosed with “lone” AF carries a much better prognosis regarding major adverse outcomes than does AF associated with traditional risk-contributing comorbidities (and despite the fact that “lone” AF patients are generally not anticoagulated).^2–5^

Importantly, the above-listed major adverse outcome events can result from aging, hypertension, heart failure, diabetes, vascular disease, and other major comorbidities themselves, even in the absence of AF **(Figure 1)**. However, the likelihood of such adverse outcomes is highest in patients who have AF plus such comorbidities. Thus, AF alone is not a simple villain. Rather, comorbidities and the atrial myopathy they induce or to which they contribute are also major additional factors to reckon with in addition to the arrhythmia—and this is more so the case the longer they last.

Notably, atrial myopathy can result from each of the comorbidities (in number and severity) and/or AF itself (via AF-induced atrial tachycardic cardiomyopathic effects) **(Figure 1)**, but rarely will it reach clinical importance when the AF is “lone.”^1^ Moreover, as the listed adverse outcomes can result from comorbidities independent of AF, such events may occur in the AF patient who is rhythm-controlled, although the risk may be lower if the AF is reduced or eliminated. Thus, in clinical trials of treatments for AF, we should not expect rhythm control to have as great an effect on such adverse outcomes as it may have on symptoms such as palpitations or quality of life, although the former are the concerns of greatest importance. Moreover, we should probably not combine in composite endpoints those that result from AF alone and those that can be consequent to any associated comorbidities. And, to reduce major adverse outcomes, optimal therapy of comorbidities must be utilized in addition to treatments for AF.

Atrial myopathy in this context represents a constellation of altered (remodeling) anatomy/histopathology (inflammation, fibrosis, apoptosis, stretch, hypertrophy, and more) induced directly by the comorbidities or indirectly by their consequent adverse left ventricular (LV) dynamics and the pressures they cause.^6^ These alterations result in: (1) changes in atrial electrophysiology (electrical remodeling) that facilitate the maintenance and progression of AF, which then further contribute to the atrial pathology in a vicious circle; (2) changes in left atrial (LA) systolic function (which can alter LV hemodynamics and are most marked when atrioventricular synchrony is lost during AF); and (3) changes in LA endothelial function (which can lose its normal anti-thrombotic physiology). The latter, especially in combination with reduced LA pumping function and increased LA size, can then result in thromboembolic propensity **(Figure 1)**.

Understanding these provides the basis for the recent clinical trial data that indicate that: (1) rhythm control earlier in the course of AF can be associated with improved long-term outcomes (whether achieved with anti-arrhythmic drugs or ablation) and (2) treatment of the associated comorbidities can contribute to better control of AF, lesser AF progression, and fewer adverse outcomes. The benefits of early rhythm control have been shown in patients with recent-onset AF, despite older age,^7^ as well as in younger patients.^8^ The benefits may be greater in younger patients with fewer comorbidities and less-established atrial myopathy.^8^ Likewise, earlier control of adverse comorbidities would expectedly reduce the development and progression of any consequent atrial myopathy. Prevention has to be better than attempts at reversing atrial fibrosis, apoptosis, etc. Considerations such as these are reflected in the 2020 European Society of Cardiology guidelines for AF^9^ to a greater degree and at a higher level of importance than they are in the older 2014 American guidelines.^10^ They are best detailed in the recent *Journal of the American College of Cardiology (JACC)* state-of-the-art review by Camm et al.,^11^ where rhythm control is the preferred approach and rate control is the “strategy of last resort.”

Hence, we can no longer base our approach to AF on the observations of historically important trials performed almost 2 decades ago in which rhythm and rate control demonstrated similar mortality outcomes when using the then-available therapies (overwhelmingly amiodarone, no ablation, no dronedarone, minimal dofetilide, and different heart failure and coronary artery disease approaches) in AF patients with variable clinical demographics. Rather, we should now assess AF in patient-specific settings, where the underlying pathophysiology is evaluated using the duration of AF history, AF pattern and burden, demographics, and imaging considerations and where modern therapies for both comorbidities and AF are employed. Symptom reduction and improved quality of life are important to our AF patients, but so too are reduced strokes, heart failure, hospitalization, and death. As recent documents suggest,^10,11^ the default approach should be to treat comorbidities (“upstream therapy,”^5,12^ consider rhythm control early, and individualize care. We cannot just consider AF itself or as the primary player any longer; rather, the company it keeps imparts a dramatic role to the presentation, pathophysiology, and outcomes in our AF patients. Both AF and any associated comorbidities must be dealt with if we are to optimize patient outcomes.

## Figures and Tables

**Figure 1:**
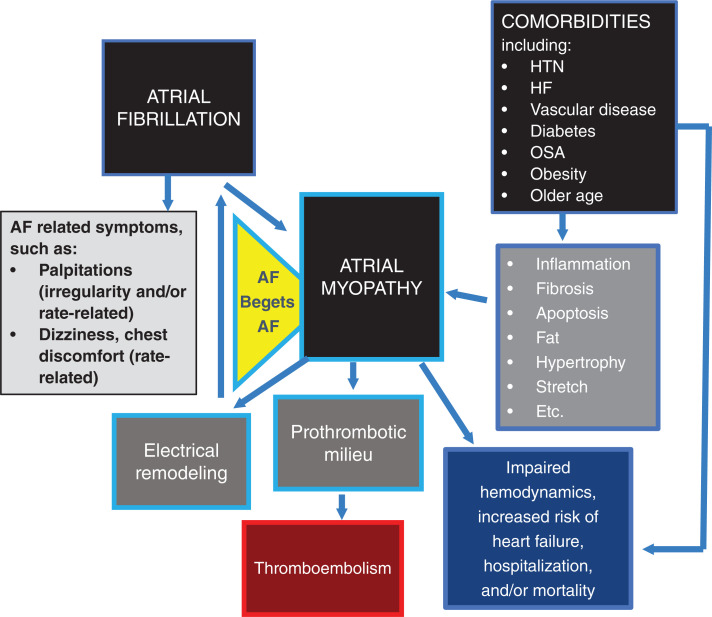
A depiction of the relationship between atrial fibrillation (AF), comorbidities, atrial myopathy, and outcomes. See text for discussion. The area in yellow depicts the AF-begets-AF loop, where AF induces atrial alterations that facilitate more AF. *Abbreviations:* AF, atrial fibrillation; HF, heart failure; HTN, hypertension; OSA, obstructive sleep apnea.
